# Farmer-Led Irrigation and Its Impacts on Smallholder Farmers’ Crop Income: Evidence from Southern Tanzania

**DOI:** 10.3390/ijerph17051512

**Published:** 2020-02-26

**Authors:** Maurice Osewe, Aijun Liu, Tim Njagi

**Affiliations:** 1 College of Economics and Management, Nanjing Agricultural University, 1 Weigang, Nanjing 210095, China; mauriceosewe@gmail.com; 2 China Center for Food Security Studies, Nanjing Agricultural University; 1 Weigang, Nanjing 210095, China; 3 Tegemeo Institute of Agricultural Policy and Development, Research, Egerton University, P.O. Box 20498-00200, Kenya; tnjagi@tegemeo.org

**Keywords:** farmer-led irrigation, household welfare, per capita crop income, smallholder, Tanzania.

## Abstract

Irrigation projects in sub-Saharan Africa are mostly unsustainable because of lack of maintenance by their users or government planners. By contrast, evidence shows that the smallholder farmers are developing and expanding the irrigated land, using their initiatives. Farmer-led irrigation, a revolutionary agricultural intensification approach, is already in progress with the magnitude to significantly transform the living standards of smallholder farmers. However, a rigorous assessment of its impact on household welfare to ascertain this is lacking. This paper bridges this gap by assessing factors influencing the adoption of this particular approach as well as its effects on the farmers’ per capita net crop income. Our data set consists of 608 smallholder farmers in Southern Tanzania and used propensity score matching to estimate the effects of adoption on the per capita net crop income. Our results indicate that the uptake of farmer-led irrigation practices is influenced by drought experience, water user group membership, farmer organization membership, and government extension, as well as the sex of the household head. Further, there was a positive and significant effect on the adopters’ per capita net crop income, thus encouraging the need to promote farmer-led irrigation as a complement to externally promoted innovations in achieving sustainable food security. This study, therefore, recommends that the government should support the farmers’ initiative by improving roads, removing market barriers, and helping farmers who have not yet taken up the initiative. Also, the government should enact regulations to make sure farmer-led irrigation initiatives do not harm the eco-environment such as protecting domestic water users. Finally, the government should leverage microservices to the farmers such as promoting affordable and appropriate credit facilities. It is necessary to continue pursuing this vein of research to gain information regarding the definite impact of the farmer-led irrigation on household welfare.

## 1. Introduction

Smallholder farmers are resuming their position as a primary focus for development after years of neglect [1]. This reflects on a broad global consensus that land, soil, and water are components of an essential emerging nexus of issues facing global demographics. Similarly, food demands are projected to increase in 2050 by 60% to sustain an ever-growing population of 9 billion [2]. In most developing economies, small-scale farming is the main farming method, yet its productivity fails to match its promising impact because of inaccessibility and rights to irrigation water. Focusing on sub-Saharan Africa, agriculture is the most significant sector and is hit the hardest by climate change [3,4]. These changes result in considerable welfare losses, particularly for the smallholder farmers whose main source of livelihood is agriculture. Hence, there is a prerequisite to defuse the potential adverse effects on farmers’ welfare. Climate adaptation seems to be an essential technique for farmers to diminish these unfavorable effects of climate change [5]. It can be achieved by implementing policies geared towards promoting appropriate and effective approaches and also farmers themselves taking adaptive measures. Previous studies indicate that farmers’ initiatives are the most applied adaptive approaches in sub-Saharan Africa. Other studies have documented several institutional, socioeconomic, and environmental factors as the fundamental factors facilitating farmers’ choice of particular farming practices in sub-Saharan Africa (SSA) [6,7,8]. However, a renewed push has equated the power of farmers’ farming initiatives as fundamental variables to transform and mitigate the impacts of climate change [9,10]. In this case, farmer-led irrigation has been a necessary means to adapt and facilitate sustainable farming productivity [11,12]. Thus, farmer-led irrigation as a method is defined as farmers having the sole decision about farming design and purpose, and technique, effecting changes in investment patterns, innovations, and market linkages as well as sustainable use of land and water [1,13]. Even more, large-scale public irrigation schemes have often failed to improve smallholder welfare, thus making the governments wary of further investments. Further, most irrigation projects were costly and ran into problems, failing to irrigate as much land as prospected. Studies about farmer-led irrigation practices in sub-Saharan Africa indicate that irrigated agricultural land is much higher than the official government evaluation reports have documented [1,14]. Farmer-led irrigation is attributed to both individual and combined farmers’ efforts, rather than small-scale and large-scale irrigation projects implemented by the government. As concluded by Khatri-Chhetri et al. [15], farmer-led irrigation facilitates the maximum use of scarce smallholder resources, and thus is a sufficient means to sustainable economic development. This approach is more of a participatory paradigm that observes smallholder farmers as innovators, growers, and caretakers of food and environment. The difference between farmer-led irrigation and the government-initiated irrigation is that farmers decide how they organize themselves and what to farm where and when, as well as which markets to sell their produce at [16]. The government-led irrigation schemes sometimes contribute to farmers losing their capacity to innovate, while creating a feeling of dependency on people outside their community.

**Figure 1:**
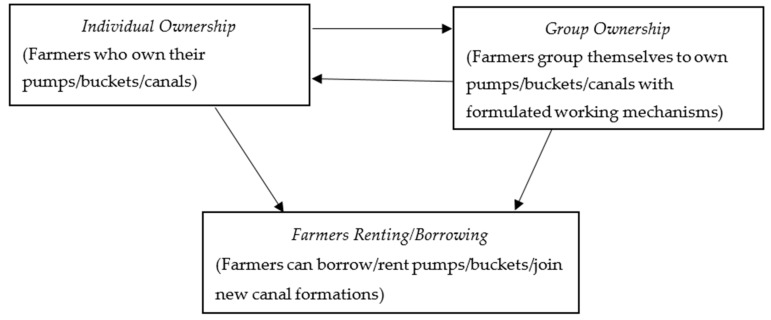
Farmer-led irrigation organization.

Agriculture is the backbone and the principal sector of the economy in Tanzania. Its performance creates a substantial effect on food security as well as poverty and income [16]. Food Agricultural Organization (FAO) [2] reported that agriculture accounts for 75% of the rural household income, 45% of the Gross Domestic Product (GDP), and 80% of employment in Tanzania. Compared to other sectors, agriculture indirectly induces economic growth through enormous consumption associations. In Tanzania, there are three distinct farmer-led irrigation practices, namely, petrol pump irrigation, bucket irrigation, and furrow irrigation. These techniques have emerged as the predominant farming methods that smallholder farmers have used to draw water from lakes, rivers, and wells to facilitate intensive horticultural production in the past decade. Particularly, furrow irrigation entails constructing and maintaining informal water canals. The first constructors are the initial owners and they take control of the water maintenance and distribution among member farmers. However, new construction or improvement of the canals attracts new users or a reconfiguration of rules and ownership. On the other hand, petrol pumps and water buckets are shared among farmers through borrowing, renting, or purchasing a new set. Also, farmers group themselves to buy new ones with ground rules on how to share and use the pump/buckets. Figure 1 explains the rationale for farmers developing, maintaining, and running this particular practice by themselves. Moreover, this agricultural practice is locally centralized and horizontal. This is typically an improved mode of farming compared to the top-down government-initiated projects. Further, farmer-led irrigation is region specific.

Currently, there is a precise demand for better institutionalization of effects evaluation and culture with a broad consideration of the intricacies of the relations between improved farmer-led irrigation and smallholder farmers’ welfare. Hence, this paper evaluates the effects of farmer-led irrigation practices on the smallholder farmers’ per capita net crop income as a proxy for improved living standards in southern Tanzania. As mentioned earlier, three farmer-led irrigation practices are considered, that is, petrol pump irrigation, bucket irrigation, and furrow irrigation. Further, the fundamental research questions that this study asked included: (i) What factors determine the adoption of farmer-led irrigation? and (ii) What are the effects of farmer-led irrigation on the smallholder farmers’ per capita net crop income? Significantly, this study focuses on enriching the literature by offering a micro-outlook on the smallholder household welfare effects of farmer-led irrigation. Assessing the impact of farmer-led irrigation adoption assists with documenting priorities, responding to survey programs, and guiding policy makers and those involved in improving farming methods dissemination to have a better understanding of how practices are integrated and spread into farming households, and offers proof that farmers gain from the survey outcomes [11].

## 2. Summary of the Literature Review

There are not many research studies on farmer-led irrigation practices because it is a new wave of research path. However, among the available literature, Woodhouse et al. [1] argued that there is an urge to review the contemporary dynamics of sub-Saharan Africa irrigation systems, particularly the roles and influence of the smallholder farmers in expanding the irrigated land. Mdee and Harrison [16] also argued that the government has not sufficiently documented farmer-led irrigation, and the current attention and magnitude of the government irrigation schemes confirm that this farming practice is happening outside the formal government framework in Tanzania. On the other hand, Xie et al. [17] observed a possible profitable smallholder irrigation expansion in sub-Saharan Africa. As a result, between 113 and 369 million rural farmers can gain from the innovations in the region, generating a net income of US $14–22 billion annually based on the fact that the farmers themselves initiate and drive the technology [17]. Farmer-led irrigation is part of the climate-smart agricultural technologies that are geared towards sustainable food security and environmental improvements in the face of climate change. On the same note, Brussow et al. [18] argued that the implementation of different climate-smart practices by farmers improved the food accessibility, availability, security, diversification, and stability in Tanzania. Further, in a study conducted in Mozambique, Beekam et al. [14] illustrated that the under-representation of the informal small-scale irrigation in Manica confirmed that farmers’ initiatives are barely recognized.

Woodhouse [19] observed a contrasting understanding of sub-Saharan Africa irrigation development. One part represents an innovation pattern that supports subsistence farming linear development to innovative farming influenced by the adoption of new practices. Also, there is a conventional perspective that presents farmers as caretakers of their environmental requirements that should not embrace change. Both paths result in a misinterpretation of the certainties through which farmers operate in Africa [20]. Thus, a new outlook is necessary to explain why smallholder farmers in sub-Saharan Africa are already venturing into farmer-led irrigation practices. Moreover, Veldwisch et al. [21] argued that farming practices initiated by farmers often increase the agricultural productivity and improve farmers’ living standards compared to the state-owned projects that perform poorly in informal setups. Informal smallholder irrigation practices have been progressively growing even though there is almost zero formal investment injected into such initiatives. According to Morris, [22,23,24], farmer-led irrigation is deemed unproductive and disregarded as spontaneous by most policymakers. On the other hand, several studies on irrigation and economic development indicate that much of the ongoing investment is proceeding without adequate consideration of what needs to be done differently to ensure irrigation projects are implemented and managed to improve livelihoods and the ecosystem [10]. Theoretically, an irrigation system can facilitate positive shifts for smallholder farmers. However, farmer-led irrigation practices offer several important benefits over centralized irrigation infrastructures. As documented by [16], farmer-led irrigation initiatives have lower unit cost and better performance outcomes than the government-supported irrigation projects. Further supporting the economic case for farmer-led irrigation, a study [20] estimated the profitable expansion potential for both the large-scale government projects and smallholder farmer irrigation. The latter exhibited higher profits as well as internal rate of return. Concluding on the literature, the farmer-led irrigation initiative is a fast-growing farming method in sub-Saharan Africa and covers a greater percentage of the irrigated land than what the official statistics indicate [19,25,26,27]. Therefore, the presence of large and rising informally irrigated lands presents sufficient opportunity to improve the smallholder farmers’ welfare, particularly in southern Tanzania. 

## 3. Materials and Methods

### 3.1. Study Area

Southern Tanzania is known for many streams that act as a source of water for agricultural farming. We conducted a survey in two districts, Kilolo and Mbarali, that lie within the Southern Agricultural Growth Corridor of Tanzania (SAGCOT). This region acts as an agricultural hub for the government as well as nongovernmental organizations. Most of the large-scale and small-scale irrigation projects have been tried in this region by the government, hence making it a potential area to test our hypothesis about the effects of farmer-led irrigation initiatives and the smallholder farmers’ welfare. Even more, this region promotes profitable agricultural farming with major benefits for smallholder farmers and local communities. Both the districts predominate in the horticultural farming system. Approximately 2 million hectares are covered by small-scale farming out of the total of 7.5 million hectares of land in the SAGCOT region. Most farmers in this region practice mixed farming methods. Kilolo is a male-dominated district with 51.5% males, and 94.6% of its total population live in rural areas. It also covers an area of 9244 km^2^ and has a population density of 23.60/km^2^. On the other hand, Mbarali also is a male-dominated district with 52.5% males, and most of its population, 71.8%, reside in the rural areas. Further, it has an area of 14,439 km^2^ and a population density of 20.81/km^2^.

### 3.2. Sampling 

This study utilized cross-sectional data collected by CIAT (International Center for Tropical Agriculture) in 2014/2015 [28]. This dataset aimed to assess smallholder farmers’ agricultural productivity as well as the intrahousehold decision-making in Mbarali and Kilolo districts in southern Tanzania. Two stages of sampling were used, where the first stage consisted of selecting the districts purposively. Further, Kilolo and Mbarali were selected because they lie at the center of the southern agricultural corridor. In the second stage, we used a proportionate random sampling method to select 608 smallholder farmers from the districts. The questionnaire captured information regarding household-specific factors, labor characteristics, farming practices, institutional characteristics, climatic characteristics, and economic factors.

### 3.3. Variable Specifications

Farmer-led irrigation increases the smallholder farmers’ crop income and irrigated land, and provide an alternative to the vulnerable rainfall in southern Tanzania. Our study evaluated the effects it has on smallholder farmers’ welfare, that is, using per capita crop income as an outcome variable for improved welfare. Further, we divided the explanatory variables into household socioeconomic characteristics, economic characteristics, institutional characteristics, and climate changes and experiences. The household socioeconomic variables included age of household head, sex of the household head, size of the household, number of years residing in the village, and household literacy index. Economic characteristics included asset index as a measure of household wealth. Institutional characteristics included membership to agricultural groups, water user groups, farmer organizations, credit services, nongovernmental organization information, and extension services. Also, climatic shocks included future climatic changes and drought experience.

### 3.4. Calculating Per Capita Net Crop Income

We conducted our survey on the household level, such that all the income generated from the crop farms in a household were summed up as gross crop income. We took note of the average sales that farmers made during harvest and after harvest. Similarly, the unsold produce was valued at the current market price to ascertain their monetary value. This formed the total gross crop income of a particular household. On the other hand, the total cost of production per farm included the variables’ costs such as labor costs, seeds, fertilizers, and transportation costs. The crop income for a particular farm was computed by including the total costs from gross income. Further, per capita net crop income was calculated as a summation of net crop income divided by the household size.

### 3.5. Empirical Model

Previous studies have cited the Propensity Score Matching (PSM) method to solve the selection bias in observational datasets [29]. As a result, this study utilized the PSM method to overcome the bias created by self-selection into farmer-led irrigation. The first research question was to determine the factors influencing the uptake of farmer-led irrigation in southern Tanzania. This was evaluated using a binary choice logistic regression method where adoption of the farmer-led irrigation was the dependent treatment variable. It formed the first step of the PSM model. In this stage, the binary logistics regression method locates the adopter and nonadopter groups that contain similar relevant observations. The binary choice model is specified as follows:

(1) $$W_{it}=\beta x_{i}+\varepsilon_{i}$$

(2) $$W_{it}=1,\;if\;W_{it}>0;0,\;otherwise$$

where, $W_{it}=1$ represents the adopter of farmer-led irrigation and $W_{it}=0$ represents the nonadopters of farmer-led irrigation. Similarly, β represents the coefficients of the model. We benchmarked our exogenous variables selection on the available literature about agricultural technologies’ adoption [30,31,32]. This first step of this analysis generated factors influencing the adoption of these farmer-led irrigation as well as the propensity scores.

In step two, the matching algorithms formed a control group that contained similarly observed variables as the treated group. The nearest neighbor matching method, radius matching method, and kernel matching algorithms were used and compared. The average treatment effect on the treated is specified mathematically as [29]:

E (Y_1_ − Y_0_|D = 1) = E (Y_1_|D = 1) − E (Y_0_|D = 1)

where, Y_1_ denotes the outcome of the *i_th_* farmer if they adopted the initiatives, Y_0_ denotes the outcome of the *i_th_* farmer if they did not adopt the initiative, and D = (0, 1) denotes the actual treatment on the *i_th_* farmer. Similarly, Y_i_ = Y_0i_ + D_i_ (Y_1i_ − Y_0i_) represents the actual perceived outcome of the *i_th_* farmer. Also, we generated a counterfactual of E (Y_0_|D =1) since it cannot be perceived directly, which is the outcome the farmers could have attained had they adopted the initiative. The matching was conducted on the common support region in line with the PSM assumptions. We further used t-test to determine the variations in the outcome variable between the matched pairs. Some studies have criticized PSM for not accounting for the unobservable variables during estimations. To solve for this, we adopted a sensitivity analysis test recommended by Rosenbaum [29]. 

## 4. Results and Discussion

### 4.1. Household Characteristics

Table 1 represents the descriptive statistics of the adopters and the nonadopters of farmer-led irrigation. The last column shows the results (*p*-values) of independent sample t-test. Values less than 0.05 indicated a significant difference row variable between the treated and the control groups. Similarly, the adopters had a slightly younger mean age compared to their counterparts. In contrast, the adopters illustrated a larger household size, literacy index, asset index, extension services, future climatic changes, drought experience, and water user groups mean than the nonadopters. For instance, larger household size could mean available labor that could assist on the farm. Similarly, the extension services were higher in adopting farmers than nonadopters. However, it is necessary to note that the extension services were basically on the farm input technologies rather than advising on the uptake of farmer-led irrigation.

### 4.2. Adoption of Farmer-Led Irrigation Practices

Our first research question sought to determine factors that influence the adoption of farmer-led irrigation. We applied the binary logistic model where the dependent variable was the binary farmer-led irrigation adoption. This evaluated the household’s propensity to adopt the initiative. We also applied the Hosmer–Lemeshow test and Variance Inflation Factor (VIF) to assess the model’s goodness of fit and multicollinearity within variables, respectively. The result indicated a correctly specified model and zero multicollinearity among the variables. Moreover, we obtained a significant log-likelihood ratio of −333.63474 at the 1% level and a pseudo R^2^ of about 0.1639. This illustrated the data were well fitted as well as the overall model used. Table 2 shows the variables that influenced farmers to adopt their own initiatives (farmer-led irrigation practices). As presented, the experience of drought, gender, asset index, water user group membership, and extension services influenced the smallholder farmers’ decision to adopt the farmer-led irrigation. However, membership to a farmer organization influenced the decision to adopt negatively. As a result, the marginal coefficients illustrated a unit change and its corresponding effects to adopt the farmer’s initiative. The findings of our research concurred with the previous literature on the factors influencing the adoption of agricultural technologies. For instance, our results indicated a positive and significant household gender variable in farmer-led irrigation adoption. Male-headed households were 9.45% more ready to adopt the farmer-led irrigation compared to female-headed households. Gender affects the uptake of new technologies since the household head is the primary decision maker. Similarly, men have more access to and authority over essential production resources compared to women in society [33,34]. Our findings are consistent with the results of Obisesan [35] that depicted male farmers as more likely to adopt new agricultural technologies than female farmers. Nonetheless, other researchers registered a mixed reaction on the roles men and women play in the case of new technology uptake [34,36]. Also, Moriss and Doss [37] observed no significant relationship between sex of the household head and improved maize adoption in Ghana. Further, they argued that rather than household head gender, technology adoption is motivated by resource availability.

The asset index is used as a proxy to estimate the smallholder’s household wealth. Households that own more assets are likely to have the resources necessary to adopt the agricultural practice. Our results indicated that a unit increment in the asset index influenced the adoption of farmer-led irrigation by 2.55%. This was expected as our results conformed with the results of Genius et al. [38] that documented a positive association between the asset index and the improved wheat varieties adoption in Ethiopia. Also, Namra et al. [39] observed that young and middle-aged male farmers were the main adopters of the current agricultural innovations. Also, being a member of a water user group affected the adoption of farmer-led irrigation positively. The outcome indicated that farmers who were a member of a water user group were 30.9% more likely to adopt. It is plausible because membership to a social group enhances social trust, information, capital, and idea exchange [7,34]. Farmer members educate and learn from each other the advantages and methods of water conservation and the benefits of irrigating their farms. This was in agreement with Uaiene et al. [40] who opined that social network impacts influence individual farmer choices, and in the agricultural innovations’ framework, farmers are free to discuss ideas among themselves. Further, Shiferaw et al. [41] observed that farmers who contribute to community-based groups are more likely to participate in information sharing and awareness about new and emerging technologies. This improves their technology uptake.

The significance of the drought experience variable indicated that farmers who had experienced drought are aware of its adverse impacts and would, consequently, welcome farming practices that are resilient, increase productivity, and enhance food security, compared to the nonadopters. The extension officers are the conduit between researchers and consumers of every technology. They reduce the transaction cost of transferring information from one farmer to the other. Our results noted that the smallholder farmers who received extension services were 6.45% likely to use farmer-led irrigation practices. The likelihood of the officers advising farmers on crop management systems rather than on the farmer-led irrigation practices can be supported by the conclusions of Mdee and Harrison [16]. They noted that farmer-led irrigation practices are not currently well recognized and the current focus on the public–private irrigation schemes only proves that this practice is extensively occurring outside the formal governance mechanisms. Other researchers also reported a direct relationship between extension services and adoption of agricultural technologies [33,40,42]. In the same breadth, Khonje et al. [34] and Simtowe [43] argued that the influence of the extension agents could equipoise the diverse effects of lack of formal education on the farmer’s choice to adopt agricultural technologies.

However, being a member of a farmer organization influenced the adoption of farmer-led irrigation negatively. Results in Table 2 show that farmers who were members of a farming organization were 14.04% less likely to adopt the practice. Bandiera and Rasul [44] argued that implementation externalities produce opposite impacts in that the more other farmers engage in testing the new farming idea, the more significant it is to accept it, but also the more advantageous it is to free-ride on others’ experimentation. Rosenzweig and Parry [45] noted that social networks increase the probability of technological uptake, but also most farmers seem to joyride on their neighbor’s costly experimentations. Hence, we concluded that membership to a farmer organization had adverse effects because most farmers were afraid to try new agricultural practices such as farmer-led irrigation practices.

### 4.3. Estimating the Effects of Farmer-Led Irrigation on Smallholder Farmers’ Crop Income 

Within the PSM framework, we used three matching methods: nearest neighbor matching, radius matching, and kernel-based matching to estimate the Average Treatment Effects on the treated (ATE). We computed ATE after matching as illustrated in Table 3. Further, the propensity score ranged between 1 and 0 for both the treated and control groups. We present the results of the three matching algorithms in Table 3. From the results, we deduced that the adoption of farmer-led irrigation creates a significant positive effect on smallholder farmers’ per capita net crop income. This is consistent with several studies on the impacts of agricultural technologies on productivity and welfare [46,47,48,49,50,51]. The three matching methods displayed consistent results; thus, the adopters had a higher per capita net crop income compared to the matched nonadopters. Assuming that the treated and the control groups were matched on equal propensities, and based on the findings, we conclude that the variations in per capita net crop income are a result of adopting farmer-led irrigation. Similarly, the significant positive effects of this initiative provide strong implications for the smallholder farmers as well as the development researchers since small-scale farming is the predominant farming method in Tanzania and other sub-Saharan countries. Hence, any improvement in crop income leads to a substantial improvement of the farmers’ food accessibility, availability, and security.

Moreover, the balancing test results in Table 4 indicated reduced pseudo R^2^ values and insignificant likelihood tests’ *p*-values. This confirmed the application of the PSM treatment estimation method for this research. Insignificance meant that there were little or no variations between the independent variable values for the treated and control groups after matching. Further, a low value of both mean and median bias validated the application and use of the propensity score matching. 

## 5. Sensitivity Analysis

**Figure 2:**
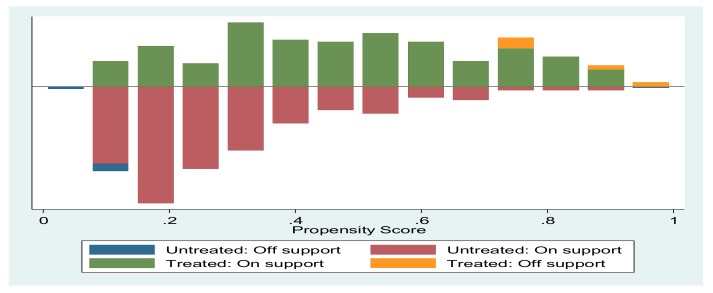
Common support graphical representation.

Several researchers have argued and noted the necessity of testing the reliability of the PSM estimations. This way, it helps researchers to understand how sensitive the estimates are based on the small propensity score deviations. Similarly, sensitivity analysis checks on the quality of matched clusters are necessary. And finally, this analysis assesses the effects of the unobserved variations on ATE and ATT values. Therefore, we statistically computed for Rosenbaum bounds sensitivity analysis and the results are presented in Table 5. The significance level was not affected even after increasing gamma values threefold. Hence, we concluded that no external deviation can change the estimated ATE and ATT values. Similarly, Figure 2 illustrates the PSM common support and distribution.

## 6. Conclusions 

The emergence of farmer-led irrigation has initiated a recent momentum around smallholder irrigation as a development priority. Perhaps notably, the World Bank and FAO are leading specific research projects with the aim of understanding the wider potential of this farmers’ initiative in terms of welfare change and water and environmental management solutions. Smallholder farmers have developed an effective irrigation approach, assisted by small affordable pumps and buckets, broadly in response to the opportunity to sell high-value agricultural products, such as vegetables, to the growing towns and localities. Similarly, several studies have outlined the prospected potentials of the farmer-led irrigation to improve the smallholder farmer’s livelihood. However, most of the research concentrates more on the coverage and official documentation of this particular practice, leaving a gap on the actual impacts it has on smallholder farmers. As a result, this research contributes to this gap by offering an empirical analysis of its actual effects regarding the farmers’ welfare.

Therefore, this paper assessed the factors influencing the uptake of farmer-led irrigation as well as its effects on the smallholder farmers’ per capita net crop income. We found that the decision to adopt this practice is determined by whether the farmers had drought experience, were members to a water user group, owned assets, received extension services, and had membership to farmer organizations, as well as by the gender of the smallholder household head. These results were in agreement with most of the literature on the adoption of agricultural technologies. However, we noted that smallholder farmers have no access to credit services, the reason being that most of the available credit facilities are not willing to loan to smallholder farmers because of their small capital base; the borrowing interest rates are high, and in some regions, there are no credit facilities. This study also found positive and significant effects of farmer-led irrigation on the smallholder farmers’ per capita net crop income. All the three matching algorithms presented consistent outcomes of both ATE and ATT. Therefore, we could conclude that adopting farmer-led irrigation could increase the smallholder farmers’ per capita net income in the range of 76,772–91,152 Tanzanian shillings. Hence, this is a noble initiative that could assist the smallholder farmers in accessing food, increasing their incomes, empowering each other, and, above all, using the available scarce resources at their disposal.

Further, this paper contributes to the growing literature that advocates for government support of farmer-led irrigation. It is imperative to continue pursuing this web of research to gain information regarding the definite impacts of the farmer-led irrigation practices on smallholder farming households. This will help create awareness in other farmers and sub-Saharan Africa at large. However, a likely drawback in this paper regards the approach used to create the matched groups. We used logistic regression to create the propensity scores because it is the most used in the literature. However, there are other possible approaches for generating propensity scores such as the probit model, discriminant function analysis (DFA), and boosted regression trees.

Based on these findings, three policy implications that could help smallholder farmers who have yet to adopt this practice are proposed. First, the government should support farmer-led irrigation practices leading to a net benefit. In this case, the government should improve roads, remove market impediments, and assist smallholder farmers who have yet to take up the chance to irrigate. Second, the government should ensure that farmer-led irrigation practices do not harm the environment. Policies should prevent over-abstraction from catchment areas, as well as protect the rights of domestic water users. Third, the government should leverage microservices to the farmers. These include affordable credit services, building on farmers’ existing knowledge and experiences, and intensifying extension services. Even more, there are a number of women irrigators and their priorities might differ from those of the male irrigators. As a result, an empirical study to determine the impact of this farmer-led irrigation on gender empowerment would be useful in future studies. In the case of irrigation, women farmers can grow food and equally benefit from increased local employment.

## Figures and Tables

**Table 1:** Household descriptive statistics.

Variables	Adopters N = 222		Nonadopters N = 386		Sample N = 608		
	Mean	SD	Mean	SD	Mean	SD	*p*-Value
Household head age (years)	48.0	13.51	48.82	14.81	48.52	14.34	0.7277
Household size (count)	5.270	2.206	4.821	2.273	4.985	2.257	0.0131 **
Household head sex (1/0)	1.094	0.293	1.192	0.394	1.156	0.363	0.0015 **
Years in the village (years)	18.38	13.25	19.69	13.32	19.21	13.30	0.2423
Literacy index (index)	0.1114	0.1665	0.1067	0.1783	0.108	0.174	0.7486
Asset index (index)	0.4688	2.1804	0.2696	1.8168	8.35 × 10^−10^	1.988	0.000 ***
Government extension (0/1)	0.4279	0.4959	0.3135	0.4645	0.355	0.479	0.0045 **
Farmer organization (0/1)	0.2748	0.4474	0.4067	0.4919	0.359	0.480	0.0011 **
Agricultural group membership (0/1)	0.1892	0.3925	0.2047	0.4040	0.199	0.400	0.6454
Water user group membership (0/1)	0.2793	0.4497	0.0518	0.2219	0.135	0.342	0.000 ***
NGO information (0/1)	0.1306	0.3378	0.1788	0.3836	0.161	0.368	0.1202
Credit services (0/1)	0.2072	0.4062	0.2176	0.4132	0.214	0.410	0.7631
Future climatic changes	0.9640	0.1868	0.9482	0.2220	0.954	0.210	0.3715
Experienced drought	0.5135	0.5009	0.2332	0.4234	0.336	0.473	0.000***

Note: *** significant at 1%, ** significant at 5%.

**Table 2:** Determinants of adopting farmer-led irrigation.

Research Variable	ME	SE	*p*-Value	VIF
Household size (count)	0.0284	0.0344	0.410	1.14
Household head age (years)	−0.0093	0.0409	0.821	2.08
Household head sex (1/0)	0.0945 *	0.0542	0.087	1.23
Literacy index (index)	−0.0187	0.1110	0.866	1.14
Years in the village (years)	−0.0010	0.0019	0.596	2.04
Asset index (index)	0.0255 ***	0.0097	0.009	1.29
Government extension (0/1)	0.0645 *	0.0382	0.091	1.14
Farmer organization (0/1)	−0.1404 ***	0.0386	0.000	1.16
NGO information (0/1)	−0.0276	0.0520	0.596	1.16
Credit services (0/1)	−0.0485	0.0459	0.291	1.11
Water user group membership (0/1)	0.3091 ***	0.0488	0.000	1.11
Agricultural group membership (0/1)	−0.0258	0.0503	0.609	1.22
Experienced drought	0.1981 ***	0.0337	0.000	1.10
Future climatic changes	−0.0100	0.0886	0.910	1.04
Constant	−1.0851	1.6489	0.510	
Log-likelihood	−333.63474			
LR chi2 (14)	130.81			
Prob>chi2	0.0000			
Pseudo-R^2^	0.1639			
Hosmer–Lemeshow chi2 (8)Prob > chi2	4.31 0.8277			

Note: *** significant at 1%, * significant at 10%.

**Table 3:** Matching algorithms results.

Matching Algorithms	Treated (ATT)	Controls (ATT)	Difference	SE	t-Stat	ATE
RM	346,907.63	222,347.92	124,559.71	59,321.74	2.10	91,151.85
KBM	346,907.63	269,317.76	77,589.86	81,418.77	0.95	76,771.80
NNM	346,907.63	219,444.83	127,462.80	65,447.42	1.95	90727.00

Note: RM—Radius Matching, KBM—Kernel-Based Matching, NNM—Nearest Neighbor Matching.

**Table 4:** Balancing test justifying matching.

Matching Method	Pseudo-R^2^	Likelihood Ratio Chi^2^	*p* > Chi^2^	Mean Bias	Median Bias
Before matching	0.163	130.45	0.000	21.8	17.3
Radius matching	0.013	7.90	0.895	5.6	4.8
Kernel-based matching	0.025	15.03	0.376	6.6	5.1
Nearest neighbor matching	0.018	10.39	0.733	5.7	4.7

**Table 5:** Sensitivity analysis results.

Gamma	Sig+	Sig-
1	0.042058	0.042058
1.25	0.373238	0.000809
1.5	0.793916	6.9 × 10^−06^
1.75	0.963412	3.7 × 10^−08^
2	0.995877	1.4 × 10^−10^
2.25	0.999665	4.5 × 10^−13^
2.5	0.999978	1.2 × 10^−15^
2.75	0.999999	0
3	1	0

## References

[1] Woodhouse P., Veldwisch G.J., Venot J.P., Brockington D., Komakech H., Manjichi Â. (2017). African farmer-Led irrigation development: Re-Framing agricultural policy and investment?. J. Peasant Stud..

[2] FAO (2013). Climate-Smart Agriculture: Sourcebook.

[3] Deressa T.T. (2007). Measuring the Economic Impact of Climate Change on Ethiopian Agriculture: Ricardian Approach.

[4] Hassan R., Nhemachena C. (2008). Determinants of African Farmers’ Strategies for Adapting to Climate Change: Multinomial Choice Analysis. Afr. J. Agric. Resour. Econ..

[5] Füssel H.M., Klein R.J.T. (2006). Climate Change Vulnerability Assessments: An Evolution of Conceptual Thinking. Clim. Chang..

[6] Mideksa T.K. (2010). Economic and Distributional Impacts of Climate Change: The Case of Ethiopia. Glob. Environ. Chang..

[7] Deressa T.T., Hassan R.M., Ringler C., Alemu T., Yesuf M. (2009). Determinants of Farmers’ Choice of Adaptation Methods to Climate Change in the Nile Basin of Ethiopia. Glob. Environ. Chang..

[8] Bryan E., Deressa T.T., Gbetibouo G.A., Ringler C. (2009). Adaptation to Climate Change in Ethiopia and South Africa: Options and Constraints. Environ. Sci. Policy.

[9] Hall C., Dawson T.P., MacDiarmid J.I., Matthews R.B., Smith P. (2017). The impact of population growth and climate change on food security in Africa: Looking ahead to 2050. Int. J. Agric. Sustain..

[10] Kamwamba-Mtethiwa J., Namara R., De Fraiture C., Mangisoni J., Owusu E. (2012). Treadle pump irrigation in Malawi: Adoption, gender, and benefits. Irrig. Drain..

[11] Pittock J., Bjornlund H., Stirzaker R., Van Rooyen A. (2017). Communal irrigation systems in South-Eastern Africa: Findings on productivity and profitability. Int. J. Water Resour. Dev..

[12] Pavelic P., Villholth K.G., Verma S. (2013). Identifying the barriers and pathways forward for expanding the use of groundwater for irrigation in Sub-Saharan Africa. Water Int..

[13] Asfaw S., Bekele S., Simtowe F., Mekbib G.H. (2011). Agricultural technology adoption, seed access constraints, and commercialization in Ethiopia. J. Dev. Agric. Econ..

[14] Beekman W., Veldwisch G.J., Bolding A. (2014). Identifying the potential for irrigation development in Mozambique: Capitalizing on the drivers behind farmer-Led irrigation expansion. Phys. Chem. Earth Parts A/B/C.

[15] Khatri–Chhetri A., Aggarwal P.K., Joshi P.K. (2017). Farmers’ prioritization of climate-smart agriculture (CSA) technologies. Agric. Syst..

[16] Mdee A., Harrison E. (2019). Critical governance problems for farmer-Led irrigation: Isomorphic mimicry and capability traps. Water Altern..

[17] Xie H., You L., Wielgosz B., Ringler C. (2014). Estimating the potential for expanding smallholder irrigation in Sub-Saharan Africa. Agric. Water Manag..

[18] Brüssow K., Anja F., Ulrike G. (2017). Implications of climate-Smart strategy adoption by farm households for food security in Tanzania. Food Secur. Sci. Sociol. Econ. Food Prod. Access Food.

[19] Woodhouse P., Sumberg J., Thompson J. (2012). Water in African Agronomy. Contested Agronomy: Agricultural Research in a Changing World.

[20] Nkoka F., Veldwisch G.J., Bolding A. (2014). Organizational Modalities of Farmer-Led Irrigation Development in Tsangano District, Mozambique. Water Altern..

[21] Veldwisch G.J.A., Bolding A., Wester P.H. (2009). Sand in the Engine: The Travails of an Irrigated Rice Scheme in Bwanje, Valley, Malawi. J. Dev. Stud..

[22] Morris J. (1987). Irrigation as a privileged solution in African development. Dev. Policy Rev..

[23] Van Der Zaag P. (2010). Viewpoint–Water variability, soil nutrient heterogeneity, and market volatility–Why sub-Saharan Africa’s Green revolution will be location-Specific and knowledge intensive. Water Altern..

[24] Lankford B. (2009). Viewpoint–The right irrigation? Policy directions for agricultural water management in sub-Saharan Africa. Water Altern..

[25] De Fraiture C., Giordano M. (2014). Small private irrigation: A thriving but overlooked sector. Agric. Water Manag..

[26] Lankford B.A. (2005). Rural Infrastructure to Contribute to African Development: The Case of Irrigation.

[27] Giordano M., De Fraiture C., Weight E., Van der Bliek J. (2012). Water for Wealth and Food Security: Supporting Farmer-Driven Investments in Agricultural Water Management.

[28] Mwungu C.M., Mwongera C., Shikuku K.M. (2017). Survey data of intra-Household decision making and smallholder agricultural production in northern Uganda and southern Tanzania. Data Brief..

[29] Jena P.R., Stellmacher T., Grote U. (2017). Can. coffee certification schemes increase incomes of smallholder farmers? Evidence from Jinotega, Nicaragua. Environ. Dev. Sustain..

[30] Manda J., Alene A.D., Gardebroek C. (2016). Adoption and impacts of sustainable agricultural practices on maize yields and incomes: Evidence from rural Zambia. J. Agric. Econ..

[31] Thirtle C., Lin L., Piesse J. (2003). The impact of research-Led agricultural productivity growth on poverty reduction in Africa, Asia, and Latin America. World Dev..

[32] Bernard T., Taffesse A.S., Gabre-Madhin E. (2008). Impact of cooperatives on smallholders’ commercialization behavior: Evidence from Ethiopia. Agric. Econ..

[33] Mignouna B., Manyong M., Rusike J., Mutabazi S., Senkondo M. (2011). Determinants of Adopting Imazapyr-Resistant Maize Technology and its Impact on Household Income in Western Kenya. AgBioforum.

[34] Khonje M., Manda J., Alene A.D., Kassie M. (2015). Analysis of adoption and impacts of improved maize varieties in eastern Zambia. World Dev..

[35] Obisesan A. (2014). Gender Differences in Technology Adoption and Welfare Impact among Nigerian Farming Households.

[36] Asayehegn G.K., Temple L., Sanchez B. (2017). Perception of climate change and farm level adaptation choices in central Kenya. Cah. Agric..

[37] Morris M., Doss C. (1999). How does gender affect the adoption of agricultural innovations. Improved Maize Technology in Ghana: Paper Presented at the Annual Meeting.

[38] Genius M., Koundouri M., Nauges C., Tzouvelekas V. (2010). Information Transmission in Irrigation Technology Adoption and Diffusion: Social Learning, Extension Services, and Spatial Effects.

[39] Namara R.E., Horowitz L., Nyamadi B., Barry B. (2011). Irrigation Development in Ghana: Past Experiences, Emerging Opportunities, and Future Directions.

[40] Uaiene R., Arndt C., Masters W. (2009). Determinants of Agricultural Technology Adoption in Mozambique.

[41] Shiferaw B., Tesfaye K., Kassie M. (2014). Managing vulnerability to drought and enhancing livelihood resilience in sub-Saharan Africa: Technological, institutional, and policy options. Weather Clim. Extrem..

[42] Akudugu M., Guo E., Dadzie S. (2012). Adoption of Modern Agricultural Production Technologies by Farm Households in Ghana: What Factors Influence their Decisions?. J. Biol. Agric. Healthc..

[43] Simtowe F., Muange E., Munyua B. Technology awareness and adoption: The case of improved pigeon pea varieties in Kenya. Proceedings of the Presentation at the International Association of Agricultural Economists Triennial Conference.

[44] Bandiera O., Rasul I. (2002). Social Networks and Technology Adoption in Northern Mozambique.

[45] Rosenzweig C., Parry M.L. (1994). The potential impact of climate change on world food supply. Nature.

[46] Alene A.D., Coulibaly O. (2009). The impact of agricultural research on productivity and poverty in sub-Saharan Africa. Food Policy.

[47] Bjornlund H., Pittock J. (2017). Exploring the productivity and profitability of small-Scale communal irrigation systems in sub-Saharan Africa. Int. J. Water Resour. Dev..

[48] Ji C., Jin S., Wang H., Ye C. (2019). Estimating the effects of cooperative membership on farmers’ safe production behaviors: Evidence from the pig sector in China. Food Policy.

[49] Udoh E.J., Titus O.B. (2008). Improved rice variety adoption and its welfare impact on rural farming households in the Akwa Ibom State of Nigeria. J. New Seeds.

[50] Mathenge M.K., Smale M., Olwande J. (2014). The impacts of hybrid maize seed on the welfare of farming households in Kenya. Food Policy.

[51] Pavelic P., Villholth K.G., Shu Y., Rebelo L.M., Smakhtin V. (2013). Smallholder groundwater irrigation in Sub-Saharan Africa: Country-Level estimates of development potential. Water Int..

